# Urolithin A in Central Nervous System Disorders: Therapeutic Applications and Challenges

**DOI:** 10.3390/biomedicines13071553

**Published:** 2025-06-25

**Authors:** Qiang Zhang, Wan Zhang, Xinya Yuan, Xiaohong Peng, Guangyuan Hu

**Affiliations:** Department of Oncology, Tongji Hospital, Tongji Medical College, Huazhong University of Science and Technology, Wuhan 430030, China; zqdoctor1222@163.com (Q.Z.); u202110363@hust.edu.cn (W.Z.); ciea@foxmail.com (X.Y.)

**Keywords:** urolithin A, central nervous system disorders, neuroprotection, mitochondria

## Abstract

With the global trend of population aging becoming increasingly pronounced, the incidence of central nervous system (CNS) disorders continues to rise, posing a significant challenge to public health systems worldwide. Currently, many CNS disorders lack effective treatments, prompting researchers to investigate the therapeutic potential of natural compounds. Urolithin A (UA), a gut microbiota-derived metabolite of ellagitannins and ellagic acid, can cross the blood–brain barrier and exhibits a favorable safety profile. This review summarizes the biosynthesis, pharmacokinetic profile, and key biological effects of UA, including its promotion of mitophagy and mitochondrial homeostasis, as well as its anti-inflammatory, antioxidant, anti-senescence, and anti-apoptotic properties. We comprehensively summarize the preclinical evidence demonstrating UA’s therapeutic potential in CNS disorders, such as Alzheimer’s disease, Parkinson’s disease, and stroke. Recent clinical trials involving UA are presented, followed by a thorough analysis of the challenges associated with translating UA-based interventions into clinical practice for CNS disorders. This work aims to support the development of UA-based therapies to improve patient outcomes and address the growing global burden of CNS disorders.

## 1. Introduction

Central nervous system (CNS) disorders represent a significant global public health challenge. According to the World Health Organization, approximately one in five individuals worldwide is affected by a CNS condition [1]. Neurodegenerative diseases, such as Alzheimer’s disease (AD) and Parkinson’s disease (PD), exhibit a marked increase in prevalence with advancing age, a trend that aligns with the ongoing global aging population [2,3]. For instance, PD alone affects up to 3% of individuals over 65, with projections indicating a 50% rise in its prevalence by 2030 [4]. Stroke, recognized as the second leading cause of death and the foremost contributor to disability globally, continues to rise in incidence, particularly in developing countries [5,6]. Additionally, traumatic CNS injuries impact millions annually [7], while CNS tumors accounted for approximately 7.7 million disability-adjusted life years in 2016, according to the Global Burden of Disease Study [8].

Urolithin A (UA) is a natural compound produced through a multi-step metabolic process by gut microbiota, derived from dietary precursors such as ellagitannins (ETs) and ellagic acid (EA). These polyphenols are abundant in foods like pomegranates, berries (such as blackberries, raspberries, and strawberries), nuts, tropical fruits (such as Camu-Camu and jaboticaba), and tea [9,10]. Chemically, UA belongs to the dibenzo[b,d]pyran-6-one derivatives, characterized by a distinct hydroxyl substitution pattern that underpins its unique metabolic properties and biological effects [11]. Extensive preclinical research highlights UA’s diverse biological effects, including anti-inflammatory, antioxidant, anti-senescence, anti-apoptotic, and promoting mitophagy [4,9,12,13]. Randomized clinical studies further validate UA’s ability to upregulate proteins linked to mitophagy and oxidative phosphorylation (OXPHOS) in muscle tissue while reducing plasma inflammatory markers, such as C-reactive protein (CRP) [14,15]. Regarding safety, clinical trials have confirmed UA’s tolerability at doses up to 1000 mg daily, with no serious adverse effects reported in interventions lasting up to four months. Notably, UA is the first compound shown in human trials to induce mitochondrial-related gene expression without significant side effects [14,15,16]. The U.S. FDA has granted UA Generally Recognized as Safe status (GRN No. 791) as a food additive.

UA has been extensively investigated in preclinical models of various CNS disorders. This review systematically integrates preclinical evidence for UA’s therapeutic potential in CNS disorders and elucidates its biosynthesis, pharmacokinetic properties, key bioactivities, and recent clinical trials involving UA. More importantly, this review provides an in-depth analysis of the challenges encountered in the clinical translation of UA for the treatment of CNS disorders.

## 2. Biosynthesis and Pharmacokinetics of Urolithin A

**Figure 1:**
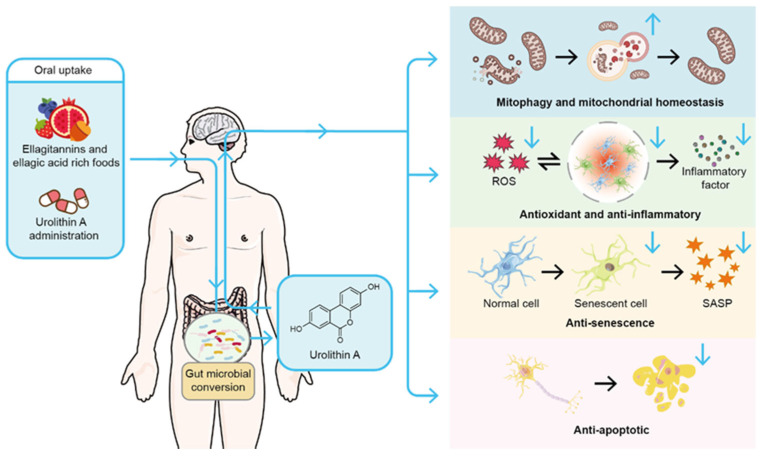
Biosynthesis and biological effects of Urolithin A. Urolithin A (UA) originates from dietary ellagitannins and ellagic acid, polyphenolic compounds prevalent in pomegranates, berries (such as blackberries, raspberries, and strawberries), and nuts. These precursors undergo stepwise metabolism by gut microbiota, resulting in the production of UA. Following its formation, UA is absorbed by the intestine, enters the bloodstream, and can cross the blood–brain barrier. Alternatively, UA may be directly ingested through oral administration. Once in the body, UA exhibits a range of biological activities, including the promotion of mitophagy and maintenance of mitochondrial homeostasis, as well as anti-inflammatory, antioxidant, anti-senescence, and anti-apoptotic effects. Blue ↑: indicates activation; blue ↓: indicates inhibition.

Following ingestion of ETs-rich food, ETs are hydrolyzed to water-insoluble EA in the stomach and small intestine. Subsequently, EA undergoes stepwise metabolism by gut microbiota in the colon to produce UA, which is then absorbed into systemic circulation (Figure 1). Specifically, the process begins with EA losing a lactone ring to form pentahydroxy-urolithins (urolithin M5). Pentahydroxy-urolithins then lose one hydroxyl group to generate tetrahydroxy-urolithins (urolithin D, urolithin E, urolithin M6, and urolithin M6R), which are subsequently metabolized into trihydroxy-urolithins (urolithin C, urolithin CR, urolithin M7, and urolithin M7R). Through a dihydroxylation reaction, dihydroxy-urolithins are formed, including UA, as well as iso-urolithin A and urolithin AR. Dihydroxy-urolithins can also undergo further metabolism to become monohydroxy-urolithins (urolithin B) [4,17,18]. *Enterocloster* species and *Gordonibacter* species participate in the metabolic conversion of EA to UA in humans [11,19]. Due to differences in gut microbiota composition across populations, there is significant variation in UA production through microbial metabolism, which is influenced by factors such as age and diet [20]. Based on urolithin production patterns, three urolithin metabotypes associated with three different urolithin production profiles have been described in human populations: UM-A (producing only UA), UM-B (producing UA, iso-urolithin A and urolithin B), and UM-0 (unable to produce UA) [17]. Studies have shown that UM-0 accounts for approximately 10% in healthy Chinese populations, 6.8–25.0% in healthy Spanish populations, 26.3% in healthy Brazilian populations, and up to 60% in healthy American populations [20,21,22,23].

Nevertheless, direct oral UA supplementation can overcome insufficient UA production caused by microbiota variations and other factors. A clinical study demonstrated that compared to consuming 240 mL of pomegranate juice rich in natural UA precursor compounds, intake of 500 mg UA supplement provided significantly higher plasma UA levels. Direct UA supplementation provided uniform circulating UA levels across the entire population, addressing the variations in endogenous UA synthesis levels due to individual differences in urolithin metabotypes (ClinicalTrials.gov: NCT04160312) [22].

Once absorbed in the intestine, UA exhibits significantly higher bioavailability than its precursor EA and can be excreted through urine and feces [18]. A portion of urolithin A undergoes further biotransformation in the liver, such as methylation, sulfation, and glucuronidation. Among these, sulfation and glucuronidation are the predominant metabolic pathways [24]. A clinical study showed that following direct oral UA supplementation, free UA and its two major metabolites, UA-glucuronide and UA-sulfate, can be detected in plasma, and the levels of UA-related metabolites are higher than those of free UA. Both free UA and its conjugated metabolites display similar kinetics, reaching peak plasma concentrations 6 h post-d Dosage. The half-lives of free UA and UA-glucuronide are 17–22 h, while that of UA-sulfate is 25–58 h, with no bioaccumulation (ClinicalTrials.gov: NCT02655393) [16]. Regarding tissue distribution, following oral administration of urolithin A in rats, UA was detected in the small intestine, colon, liver, prostate, kidneys, heart, and lungs [25,26]. To date, UA has been detected in various human tissues, including prostate, colon, breast, and skeletal muscle [16,17]. Furthermore, animal studies demonstrate that UA can cross the blood–brain barrier (BBB) in its free form. In rat models, brain UA concentrations reach approximately 10% of plasma levels following pomegranate juice consumption [27]. Mice were administered UA via intraperitoneal injection at a dose of 5 mg/kg. Subsequent mass spectrometry analysis detected free UA in both plasma and perfused brain samples. However, the concentration of UA-related metabolites, such as UA-sulfate, was much lower in brain samples than in plasma. UA-3-glucuronide was detected only in plasma [28].

## 3. Biological Effects of Urolithin A

### 3.1. Mitophagy and Mitochondrial Homeostasis

Mitophagy is a selective autophagy process that removes dysfunctional or superfluous mitochondria, thereby precisely regulating their quantity and quality to sustain cellular energy metabolism and homeostasis [29,30]. This mechanism involves multiple signaling pathways and molecules, including the well-characterized phosphatase and tensin homolog-induced putative kinase 1 (PINK1)/E3 ubiquitin-protein ligase parkin (Parkin) pathway. When mitochondria are damaged or dysfunctional, a drop in membrane potential stabilizes PINK1 on the outer mitochondrial membrane, recruiting and activating Parkin [31,32]. Activated Parkin ubiquitinates outer membrane proteins such as mitofusin 2 (MFN2), thereby tagging damaged mitochondria with ubiquitin chains [33]. These modifications recruit selective autophagy receptors such as p62 and optineurin (OPTN), which anchor mitochondria to autophagosomal membranes via associated protein 1 light chain 3 (LC3)-interacting domains, initiating engulfment [34,35]. Beyond the PINK/Parkin pathway, receptors like B-cell leukemia/lymphoma 2/adenovirus E1B 19 kDa interacting protein 3-like protein (BNIP3L) independently mediate mitochondrial clearance under specific conditions, such as during erythrocyte maturation, by directly binding LC3 [36]. Mitochondrial fission also facilitates mitophagy by generating fragmented mitochondria more readily targeted for removal, with dynamin-related protein 1 (DRP1)-mediated fission events cooperating with proteins like B-cell leukemia/lymphoma 2/adenovirus E1B 19kDa protein-interacting protein 3 (BNIP3) to selectively eliminate dysfunctional mitochondria [37,38]. Additionally, the inner membrane protein prohibitin 2 (PHB2) exposes its LC3-binding domain upon membrane potential loss, directly mediating autophagosomal recognition of mitochondria [39]. The entire mitophagy process is orchestrated through multilayered regulatory mechanisms involving mitochondrial dynamics, ubiquitin-proteasome system modifications, and metabolic signal integration, exemplified by reactive oxygen species (ROS). These coordinated processes collectively maintain homeostasis between cellular energy metabolism and damaged mitochondrial clearance [35,40].

Mitochondrial homeostasis relies on the interplay of biogenesis, fusion/fission, and mitophagy. Defects in mitophagy lead to the accumulation of dysfunctional mitochondria, triggering oxidative stress, calcium dysregulation, and energy deficits [41]. This pathological cascade is particularly pronounced in neurodegenerative disorders of the CNS. In AD, impaired mitophagy in the hippocampus and induced pluripotent stem cell-derived neurons contributes to amyloid-β (Aβ) accumulation and tau hyperphosphorylation. Pharmacological enhancement of mitophagy clears Aβ plaques and reverses cognitive deficits [41,42]. Similarly, in PD, abnormal α-synuclein aggregation—a hallmark feature—exhibits bidirectional regulation with mitophagy defects. Pathological α-synuclein suppresses key mitophagy molecules like PINK1, leading to the buildup of defective mitochondria, which in turn release ROS that exacerbate α-synuclein phosphorylation and aggregation [43].

Studies across diverse organisms—cells, *C. elegans*, mice, and humans—consistently demonstrate that UA significantly influences mitochondrial health by modulating mitophagy (Figure 1). In *C. elegans*, UA activates the PINK1/Parkin pathway, enhancing mitophagy, improving mitochondrial morphology, extending healthy lifespan, and boosting motor function [12]. In transgenic tau *C. elegans* models, UA reverses memory deficits linked to tau hyperphosphorylation by enhancing mitophagy and reducing neuroinflammatory markers [41].

In mouse disease models, UA’s role in regulating mitophagy and maintaining mitochondrial homeostasis is well validated. In AD mouse models, UA enhances microglial phagocytosis of Aβ plaques, suppresses neuroinflammation, and reduces tau hyperphosphorylation by restoring mitophagy to eliminate abnormal mitochondria [41]. In doxorubicin-induced cardiomyopathy mice, UA upregulates p62, LC3-II, PINK1, and Parkin expression, restoring impaired mitophagy, mitigating membrane potential loss and ROS accumulation, and alleviating cardiomyocyte apoptosis and mitochondrial dysfunction [44]. In Duchenne muscular dystrophy mice, UA boosts the expression of mitophagy-related proteins (PINK1, Parkin, and BNIP3), restoring muscle stem cell regeneration, improving mitochondrial respiration, and increasing survival rates [45]. Furthermore, in diabetes-associated cognitive impairment models, UA enhances PINK1/Parkin-dependent mitophagy, improving neuronal mitochondrial function and ameliorating cognitive deficits [46].

In human studies, UA’s clinical potential is increasingly evident. Randomized controlled trials in healthy middle-aged and older adults show that oral supplementation with 500–1000 mg of UA significantly improves skeletal muscle endurance and mitochondrial efficiency, reduces plasma inflammatory markers (such as C-reactive protein), and upregulates muscle proteins linked to mitophagy and oxidative phosphorylation [14,15].

These findings collectively highlight UA’s ability to regulate mitophagy and maintain mitochondrial homeostasis across species and disease models. By coordinating mitochondrial quality control networks, UA ameliorates mitochondrial dysfunction in various conditions, laying a foundation for its therapeutic application in CNS disorders.

### 3.2. Anti-Inflammatory and Antioxidant

The progression of most CNS disorders involves a complex interplay between inflammation and oxidative stress, forming a pathological network [47,48,49,50,51]. Inflammation activates microglia and astrocytes, triggering the release of pro-inflammatory cytokines and ROS. These mediators amplify local oxidative stress and impair mitochondrial function, further increasing free radical production [52]. Conversely, oxidative stress induces lipid peroxidation, DNA damage, and the release of mitochondrial DNA, which activate inflammatory signaling pathways, perpetuating a neuroinflammatory microenvironment [53,54]. This vicious cycle increases BBB permeability, promotes neuronal apoptosis, and disrupts synaptic function, ultimately accelerating CNS disease progression [47]. In AD, inflammation and oxidative stress synergistically drive Aβ accumulation and neuronal damage. Aβ buildup triggers an inflammatory response, activating microglia and exacerbating oxidative stress, which further impairs neuronal function [51]. In stroke, oxidative stress is particularly pronounced during cerebral ischemia-reperfusion injury, causing mitochondrial damage and a cytokine storm that intensifies brain tissue injury [55].

UA exhibits potent anti-inflammatory and antioxidant effects across multiple disease models, acting through diverse pathways (Figure 1). Molecular docking and dynamics simulations reveal a strong interaction between UA and human p38 mitogen-activated protein kinase (MAPK), with a binding energy of -10.1 kcal/mol, comparable to established anti-inflammatory drugs [56]. In lipopolysaccharide (LPS)-stimulated BV-2 microglia, UA suppresses the nuclear factor kappa-light-chain-enhancer of activated B cells (NF-κB) signaling pathway, reducing the release of inflammatory cytokines such as interleukin 6 (IL-6), IL-1β, and tumor necrosis factor alpha (TNF-α), while also inhibiting the phosphorylation of MAPK and serine/threonine kinase 1 (Akt), thereby halting the inflammatory cascade [57,58,59]. This effect is corroborated in LPS-induced neuroinflammatory mouse models, where UA activates sirtuin 1 (SIRT1)-mediated deacetylation of NF-κB p65, suppressing glial cell activation and the production of pro-inflammatory cytokines (IL-1β, IL-6, and TNF-α), thus preventing neuronal loss and hippocampal synaptic damage [60]. In amyloid precursor protein (APP)/presenilin 1 (PS1) transgenic AD mice, UA enhances brain adenosine 5′-monophosphate-activated protein kinase (AMPK) activation, attenuating NF-κB and MAPK activity, mitigating neuroinflammation, and supporting synaptic recovery [61]. In a renal ischemia-reperfusion injury model, UA activates the p62—kelch-like ECH-associated protein 1 (Keap1)—nuclear factor erythroid 2-related factor 2 (Nrf2) pathway, boosting superoxide dismutase and catalase activity while lowering ROS levels [62]. Similarly, in acetaminophen-induced acute liver injury, UA mitigates glutathione depletion and lipid peroxidation via the Nrf2/antioxidant response element (ARE) pathway, outperforming the clinical standard N-acetylcysteine [63]. In LPS-induced acute lung injury, UA upregulates the Keap1-Nrf2/heme oxygenase 1 (HO-1) pathway to inhibit ferroptosis and reduce lipid peroxide accumulation in lung tissue [64]. A key mechanism underlying UA’s anti-inflammatory and antioxidant effects is its regulation of mitochondrial function. In an osteoarthritis mouse model, UA enhances mitophagy in articular cartilage, reducing cartilage degradation and synovial inflammation [65]. In a sleep deprivation-induced hippocampal inflammation model, UA curbs microglial activation and mitochondrial dysfunction, lowering ROS levels and preserving neuronal morphology [66]. Recent study has revealed that age-related mitochondrial DNA (mtDNA)- cyclic GMP-AMP synthase (cGAS)/stimulator of interferon genes (STING)-mediated inflammation is physiologically upregulated across different species and organs, which can be alleviated by urolithin A-induced mitophagy [28].

### 3.3. Anti-Senescence

Cellular senescence is an irreversible state of cell cycle arrest characterized by distinct molecular and functional changes, including the loss of proliferative capacity, altered metabolic activity, and the expression of senescence-associated secretory phenotypes (SASP) [67]. In various CNS disorders, senescent brain cells persistently release SASP factors, triggering chronic neuroinflammation and exerting toxic effects on surrounding healthy cells. This process contributes to symptoms such as memory impairment and cognitive decline [28,68,69,70,71,72,73,74,75,76]. Studies suggest that neurons, under conditions of metabolic dysregulation or inflammation, may enter a senescent state by activating signaling pathways such as p21^CIP1/WAF1^, while secreting SASP factors like IL-1β, IL-6, and TNF-α, which amplify the local inflammatory microenvironment [77]. In AD, senescent microglia in mouse brains enhance NF-κB signaling via H3K18 lactylation, upregulating IL-6 and IL-8 expression, thereby intensifying neuroinflammation and amyloid plaque deposition [69]. Similarly, cellular senescence contributes to PD progression through multiple mechanisms, including SASP secretion, mitochondrial dysfunction, loss of protein homeostasis, and genomic instability [72]. Our research demonstrates that senescent pericytes disrupt the BBB, exacerbating radiation brain injury (RBI) and promoting glioma cell growth and invasion [78]. In glioblastoma mouse models, radiation-induced senescent astrocytes have been shown to enhance tumor growth and invasiveness [79].

UA, a natural metabolite, exhibits anti-senescence properties across diverse disease models through multiple pathways (Figure 1). In human microglia cell line (HMC3), UA synergizes with nicotinamide riboside to modulate immunometabolism, enhancing mitochondrial respiration and reducing DNA damage-induced senescence [80]. In quinolinic acid-induced senescence of C. elegans microglia, UA restores mitophagosome formation, reducing damaged mitochondrial accumulation, alleviating senescent phenotypes, and improving overall health [81]. In skin photoaging studies, UA activates the Nrf2/ARE pathway to mitigate UVA-induced ROS accumulation and regulates the sirtuin 3 (SIRT3)/forkhead box protein O3 (FOXO3)-PINK1/Parkin network to restore mitochondrial function, thereby ameliorating senescence in human fibroblasts [82]. In a replicative senescence model of human skin fibroblasts, UA increases type I collagen expression, reduces matrix metalloproteinase 1 (MMP-1) levels, and activates Nrf2-mediated antioxidant responses, underscoring its anti-aging effects [13]. In senescent human lung fibroblasts, UA enhances circadian rhythm amplitude via SIRT1-mediated Period circadian regulator 2 (PER2) degradation, improving cellular metabolism and mitochondrial function [83]. In auditory cell senescence models, UA treatment reduces H_2_O_2_-induced p53 and p21 expression, restores mitochondrial membrane potential and ATP synthesis, and decreases the proportion of senescent cells [84]. In cultured mouse salivary gland organoids, UA activates mitophagy to reduce damaged mitochondrial buildup, mitigating radiation-induced senescence and enhancing salivary gland stem/progenitor cell function [85]. In a cartilage degeneration model, UA treatment of mechanically stressed senescent chondrocytes increases collagen type II and aggrecan expression while lowering MMP-13 and IL-6 levels [86]. In a rat model of intervertebral disc degeneration, UA suppresses TNF-α-mediated MMP-3/13 expression and alleviates oxidative stress-induced nucleus pulposus stem cell senescence via the SIRT1/peroxisome proliferator-activated receptor gamma coactivator 1-alpha (PGC-1α) pathway [87,88]. Furthermore, in corneal epithelial cells, UA inhibits ferroptosis-related marker ferroptosis-related marker acyl-CoA synthetase long-chain family member 4 (ACSL4), upregulates glutathione peroxidase 4 (GPX4), and mitigates hyperosmotic stress-induced senescence [89]. Additionally, research has demonstrated that urolithin A corrects abnormal mitochondrial accumulation in hematopoietic stem cells of aged mice, promotes mitochondrial turnover, and thereby significantly restores energy metabolism and function of hematopoietic stem cells, ultimately enabling aged mice to recover normal hematopoietic capacity [90].

These findings highlight UA’s broad anti-senescence effects across diverse tissue systems, providing a scientific foundation for its potential therapeutic application in CNS disorders linked to cellular senescence.

### 3.4. Anti-Apoptotic

Apoptosis, a highly conserved form of programmed cell death, involves the systematic dismantling of cellular structures through a caspase-mediated cascade. This process plays an essential role in neural development and homeostasis maintenance [91]. In CNS disorders, dysregulated apoptotic pathways are a central mechanism driving neuronal degeneration. In AD, Aβ activates caspase-6, triggering aberrant tau protein cleavage, which leads to neurofibrillary tangle formation and synaptic dysfunction [92]. Pathological tau also induces aberrant cell cycle re-entry in terminally differentiated neurons, pushing them toward apoptosis [93]. In PD, α-synuclein aggregates activate the NLR family pyrin domain containing 3 (NLRP3) inflammasome, promoting caspase-1-dependent death of dopaminergic neurons [94]. Apoptosis is similarly critical in other CNS conditions. Following spinal cord injury, neuronal and glial apoptosis exacerbates inflammation and functional deficits during secondary injury phases; inhibiting caspase-3 activity or modulating B-cell lymphoma 2 (Bcl-2) family proteins markedly reduces neuronal loss and supports recovery [95,96]. In ischemic stroke models, apoptosis not only causes direct neuronal death in the infarct core but also amplifies damage in the surrounding penumbra by releasing pro-inflammatory factors [97].

UA demonstrates potent anti-apoptotic effects across diverse disease models (Figure 1). In AD studies using APP/PS1 mice, UA reduces Aβ deposition and mitigates neuronal apoptosis by downregulating tau phosphorylation and the expression of APP-related enzymes, ultimately improving cognitive function [61]. In a controlled cortical impact model of traumatic brain injury, UA suppresses the phosphatidylinositol 3-kinase (PI3K)/Akt/mammalian target of rapamycin (mTOR) and Akt/IκB kinase (IKK)/NF-κB signaling pathways, reducing neuronal apoptosis while enhancing BBB integrity and neurological outcomes [98]. In a middle cerebral artery occlusion model, UA treatment lowers Bcl-2 expression and elevates Bcl-2 associated X protein (Bax) and caspase-3 levels in the hippocampus, attenuating neuronal damage and apoptosis [99]. Beyond CNS disorders, UA’s protective role extends to other systems. In diabetes-related studies, UA activates autophagy to inhibit pancreatic β-cell apoptosis, evidenced by reduced cleaved-caspase-3 and cleaved-caspase-1 expression, an effect reversible by the autophagy inhibitor chloroquine [100]. In a model of severe acute pancreatitis-related cardiac injury, UA restores mitochondrial membrane potential and ATP production in cardiomyocytes, balancing carnitine palmitoyltransferase1-dependent fatty acid oxidation to reduce apoptosis [101]. In intervertebral disc degeneration, UA induces mitophagy via the AMPK pathway, alleviating tert-butyl hydroperoxide (TBHP)-induced mitochondrial dysfunction and intrinsic apoptosis in nucleus pulposus cells, while delaying disc structural damage and extracellular matrix degradation in a rat puncture model [102]. In acute kidney ischemia-reperfusion injury, UA activates the p62-Keap1-Nrf2 pathway to mitigate tubular epithelial cell apoptosis, improving renal function [62]. Similarly, in acetaminophen-induced liver injury, UA activates the Nrf2/ARE pathway to suppress hepatocyte apoptosis, an effect abolished by Nrf2 gene silencing [63].

### 3.5. Putative Mechanisms of Urolithin A’s Biological Effects

**Figure 2:**
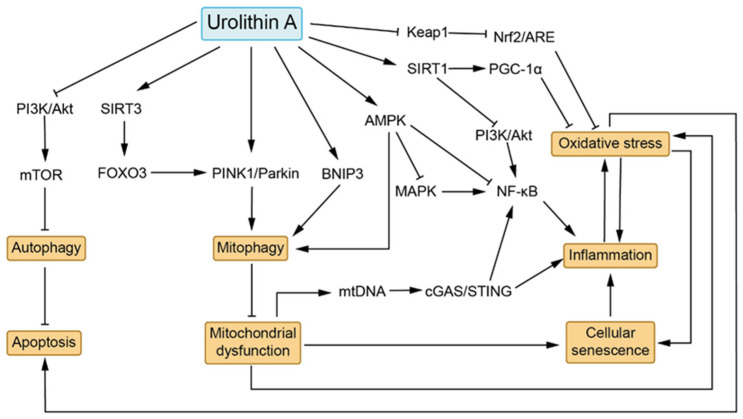
Hypothesized mechanistic network of Urolithin A. UA exerts biological activities including promotion of mitophagy, antioxidant stress, anti-inflammation, anti-senescence, and anti-apoptosis through mechanisms involving activation of PINK1/Parkin, BNIP3, AMPK, SIRT1, and SIRT3, as well as inhibition of PI3K/Akt and Keap1. Activation of PINK1/Parkin, BNIP3, and AMPK promotes mitophagy, while SIRT3/FOXO3 activation can indirectly regulate mitophagy through PINK1/Parkin pathway activation. Activated AMPK can inhibit MAPK and NF-κB pathway activity, thereby reducing inflammatory responses. SIRT1 activation and Keap1 inhibition suppress cellular oxidative stress through PGC-1α deacetylation and Nrf2/ARE pathway activation, respectively. Additionally, SIRT1 can directly inhibit the PI3K/Akt pathway, thereby suppressing NF-κB pathway activation. Mitophagy, oxidative stress, inflammation, cellular senescence, and apoptosis are closely interconnected. Mitophagy promotes clearance of dysfunctional mitochondria, reducing mtDNA release and ROS production, thereby diminishing cGAS/STING pathway and NF-κB pathway activation as well as oxidative stress. Furthermore, clearance of dysfunctional mitochondria and alleviation of oxidative stress can ameliorate cellular senescence. UA’s ameliorative effects on oxidative stress reduce the occurrence of apoptosis. UA can also promote autophagy by inhibiting PI3K/Akt/mTOR pathway activity, thereby reducing apoptosis. →: indicates activation; ┤: indicates suppression.

Based on the aforementioned research findings, we summarize the potential molecular mechanisms underlying UA regulation of various biological effects in Figure 2, constructing a mechanistic network. Specifically, UA exerts a series of biological effects through activation of PINK1/Parkin, BNIP3, AMPK, SIRT1, and SIRT3, as well as inhibition of PI3K/Akt and Keap1. Activated PINK1/Parkin, BNIP3, and AMPK can directly mediate mitophagy, clearing dysfunctional mitochondria [44,45,46]. Additionally, UA can indirectly regulate the PINK1/Parkin pathway through SIRT3/FOXO3 to promote mitophagy [82]. The clearance of dysfunctional mitochondria contributes to maintaining mitochondrial homeostasis, thereby reducing mtDNA release and ROS production, diminishing activation of the cGAS/STING pathway and NF-κB pathway, and reducing cellular oxidative stress, which consequently attenuates inflammatory responses [28,57,58,59]. Mitochondrial dysfunction and oxidative stress are closely associated with cellular senescence; clearing damaged mitochondria can alleviate cellular senescence and reduce SASP production [85]. Activated AMPK can inhibit the MAPK and NF-κB pathways, reducing pro-inflammatory factor production [61]. SIRT1 activation and Keap1 expression inhibition promote transcription of downstream antioxidant stress genes through PGC-1α deacetylation and Nrf2/ARE pathway activation, respectively, thereby alleviating cellular oxidative stress [62,63,64]. Furthermore, activated SIRT1 can also inhibit the PI3K/Akt pathway, thus reducing NF-κB pathway activation [60]. Alleviation of oxidative stress and improvement of mitochondrial function can reduce the occurrence of apoptosis [101]. UA can also inhibit the PI3K/Akt pathway, thereby blocking mTOR activation-mediated inhibition of autophagy, ultimately achieving anti-apoptotic effects [98].

## 4. Preclinical Studies of Urolithin A in CNS Disorders

### 4.1. Alzheimer’s Disease

AD is a progressive neurodegenerative disorder characterized by cognitive decline and memory impairment. Affecting over 55 million people worldwide, it accounts for 60–80% of all dementia cases, with projections estimating a rise to 139 million by 2050 [103]. The etiology of AD is multifaceted: early-onset familial AD is linked to mutations in the *APP*, *PS1*, and presenilin 2 (*PS2*) genes, while late-onset sporadic AD is strongly associated with the apolipoprotein E4 (ApoE4) genotype and aging [2,104]. Hallmark pathological features include the accumulation of Aβ plaques and neurofibrillary tangles formed by hyperphosphorylated tau protein, both of which increase with age. The progression of AD involves diverse cellular changes, such as mitochondrial dysfunction, oxidative stress, cellular senescence, synaptic damage, and neuroinflammation mediated by microglia [69,103,105,106,107,108]. Clinically, AD manifests primarily through hippocampal and cortical dysfunction, leading to spatial disorientation, language deterioration, and, in some cases, behavioral symptoms like anxiety and social withdrawal [109,110].

UA has demonstrated multifaceted therapeutic potential across various AD models. In SH-SY5Y-APP695 cell line, UA enhances mitochondrial biogenesis by upregulating the expression of Mitochondrial Transcription Factor A (TFAM) and estrogen-related receptor (ESRR) genes, although it does not significantly affect mitophagy or overall mitochondrial function in this model [111]. Further cellular studies show that UA reduces Aβ production in APPSwe-transfected human neural stem cells, while also suppressing neuronal apoptosis through enhanced autophagic flux and SIRT1 activation [112]. In Aβ42-transgenic *C. elegans*, UA induces mitophagy, reduces Aβ oligomer deposition, and mitigates memory deficits [41]. In mouse models, including APP/PS1, 3xTgAD, and those injected intracerebrally with okadaic acid, long-term UA administration markedly lowers Aβ levels in the hippocampus and cortex, reduces amyloid plaque burden, and improves spatial and learning memory [41,61,105,113,114]. These effects are mediated by enhanced microglial phagocytosis of Aβ and improved autophagy-lysosome function, which facilitates the clearance of intracellular Aβ and phosphorylated tau [41,114]. In the 5xFAD model, UA modulates the small ubiquitin-like modifier 2 (SUMO2) and ADP-ribosylation factor 5 (ARF5) protein network and suppresses overactivation of the anterior basolateral amygdala to ventral CA1 (aBLA-vCA1) circuit, significantly improving social behavior [115]. Additionally, UA activates the AMPK signaling pathway to inhibit beta-site amyloid precursor protein cleaving enzyme 1 (BACE1) and APP expression, reducing Aβ production, and lowers tau phosphorylation by inhibiting dual-specificity tyrosine-phosphorylation regulated kinase 1 (ADYRK1A) kinase activity [61,113]. Across multiple AD models, UA exhibits anti-inflammatory properties by suppressing the synthesis and release of pro-inflammatory cytokines (IL-1β, IL-6, TNF-α) and reducing the activation of microglia and astrocytes, thereby alleviating AD-related symptoms [41,61,105]. Notably, combination therapy enhances UA’s efficacy; in humanized homozygous amyloid beta knockin mice modeling late-onset AD, UA combined with green tea extract (Epigallocatechin gallate) more effectively reduces brain Aβ40 and Aβ42 levels compared to UA alone [106].

Despite UA’s promising results in AD models, most studies employ short-term interventions, leaving the long-term effects of lifelong administration on disease progression unexplored.

### 4.2. Parkinson’s Disease

PD is a progressive neurodegenerative disorder characterized by bradykinesia, resting tremor, muscle rigidity, and impaired posture and gait. Its pathological hallmarks include the gradual loss of dopaminergic neurons in the substantia nigra and the accumulation of misfolded α-synuclein into Lewy bodies [3,116]. Genetic studies have identified mutations in genes such as synuclein alpha (*SNCA*) and leucine rich repeat kinase 2 (*LRRK2*), which impair mitochondrial function, lysosomal autophagy, or α-synuclein metabolism, directly contributing to neuronal damage. Environmental factors, such as exposure to neurotoxins, may exacerbate this process by inducing oxidative stress and neuroinflammation [116]. Disease progression involves multiple mechanisms, including the trans-neuronal spread of α-synuclein, chronic inflammation driven by aberrant microglial activation, and disrupted mitochondrial energy metabolism [3].

Research across various PD models has demonstrated the multi-target neuroprotective effects of UA. In a rotenone-induced rat model, UA were detected in the brain following pomegranate juice intake, correlating with restored dopamine release and reduced α-synuclein levels [27,117]. In mice treated with 6-hydroxydopamine, UA enhanced mitochondrial biogenesis via the SIRT1/PGC-1α signaling pathway, increasing dopaminergic neuron survival in the substantia nigra and alleviating motor deficits [118]. Similarly, in a manganese-induced PD mouse model, UA promoted mitophagy, mitigating dysfunction and improving neurobehavioral outcomes while reducing harmful microglial activation [119]. In 1-Methyl-4-phenyl-1,2,3,6-tetrahydropyridine (MPTP)-treated mice, UA administration elevated striatal dopamine levels and enhanced motor coordination, accompanied by suppression of NLRP3 inflammasome activation in microglia and decreased release of pro-inflammatory cytokines such as IL-1β and TNF-α [120,121]. Furthermore, studies on the cyclin-dependent kinase 5 (CDK5)—ubiquitin-specific peptidase 30 (USP30) signaling pathway revealed that UA restores mitophagy efficiency, counteracting MPTP-induced activation of the mitochondrial antiviral signaling protein (MAVS) inflammatory pathway and offering a specific therapeutic target for toxin-induced PD [121].

### 4.3. Diabetes-Associated Cognitive Impairment

Diabetes-associated cognitive impairment is a chronic neurological complication closely linked to diabetes, characterized by a progressive decline in cognitive functions, including memory, executive function, and processing speed. Epidemiological studies indicate that type 2 diabetes increases the risk of AD by 1.5 to 2.5 times [122]. In streptozotocin (STZ)-induced diabetic mouse models, UA mitigates cognitive deficits by regulating tissue transglutaminase 2 (TGM2)-dependent endoplasmic reticulum (ER)-mitochondria contacts and calcium homeostasis, thereby reducing Aβ production and tau phosphorylation [123]. In a model of type 2 diabetes induced by a high-fat diet combined with STZ, UA treatment significantly ameliorates cognitive dysfunction. This improvement is accompanied by reduced serum levels of metabolic endotoxemia and pro-inflammatory cytokines, as well as suppression of endoplasmic reticulum (ER) stress and excessive tau phosphorylation in hippocampal neurons [46,124,125]. Furthermore, in HT22 cells, UA inhibits ER stress-mediated apoptosis by downregulating the expression of the ATPase sarcoplasmic/endoplasmic reticulum Ca2+ Transporting 3 (*Atp2a3)* gene, although overexpression of *Atp2a3* abolishes UA’s neuroprotective effects [125].

Beyond these cellular mechanisms, UA’s regulation of diabetes-associated cognitive impairment extends to the gut-brain axis. Studies demonstrate that UA improves systemic inflammation and gut barrier dysfunction by modulating the N-glycan biosynthesis pathway, an effect independent of gut microbiota or short-chain fatty acid metabolism [124]. These multifaceted molecular mechanisms—encompassing mitochondrial homeostasis, ER stress, and inflammatory responses—highlight UA as a promising therapeutic target for intervening in diabetes-associated cognitive impairment.

### 4.4. Stroke

Stroke is an acute cerebrovascular disorder caused by abnormal blood supply to the brain, primarily encompassing ischemic stroke, resulting from vessel occlusion, and hemorrhagic stroke, stemming from vessel rupture. Its onset is directly linked to cerebral ischemia and hypoxia or hematoma-induced compression of brain tissue triggered by these vascular events [5,126,127].

Preclinical studies highlight the protective potential of UA in stroke models. In mice subjected to middle cerebral artery occlusion, UA treatment significantly reduced infarct volume, improved neurological deficit scores, and ameliorated spatial memory impairments. It also suppressed neuronal apoptosis and mitigated neuroinflammation [99,128]. Mechanistic insights reveal that UA alleviates oxygen-glucose deprivation/reperfusion-induced damage in N2a cells and primary neurons, not via mitophagy, but by reducing ER stress through autophagy activation [128]. In a rat model of subarachnoid hemorrhage, UA modulated autophagy via the AMPK/mTOR pathway, decreasing cortical neuron apoptosis, enhancing BBB integrity, and reducing brain edema and neurological dysfunction [129].

### 4.5. Traumatic CNS Injuries

Traumatic CNS injuries arise from external mechanical forces impacting the brain or spinal cord, triggering a cascade of pathological events. These include secondary neurodegeneration following initial damage, characterized by neuronal death, uncontrolled inflammation, and blood–brain barrier disruption [50,130,131]. Such injuries often result in permanent disability or loss of motor and sensory functions, affecting millions globally each year. Notably, moderate-to-severe traumatic CNS injuries are linked to an elevated risk of neurodegenerative diseases [7].

In a C57BL/6J mouse model of traumatic brain injury, UA treatment reduced cortical neuron apoptosis, alleviated brain edema, suppressed inflammatory pathways, and improved neurological outcomes [98]. Similarly, in a mouse model of impact-induced spinal cord injury, UA enhanced motor function recovery. Further investigation revealed that UA inhibited pyroptosis following spinal cord injury by promoting autophagy [132]. In the SCI microenvironment, esterase overexpression is a pathological response. A study reported a novel silica-based integrated nanocarrier, prepared by incorporating carbamate-bridged UA into silica nanoparticles, called PEGylated UA-silicon hybrid nanoparticles (PUASi NPs). PUASi NPs can be specifically recognized and cleaved by esterase to release UA, thereby achieving targeted lesion delivery and enhancing UA’s efficacy in treating SCI via its ferroptosis-inhibiting and anti-inflammatory effects [133].

### 4.6. CNS Infectious Diseases

Initial research on UA in the context of CNS infectious diseases has yielded promising results. In a human neuroblastoma cell model (SK-N-SH cell line) infected with the Enterovirus 71 (EV71) virus, UA significantly inhibited viral replication, demonstrating superior antiviral effects compared to the conventional drug ribavirin. This suggests its potential for application in the treatment of aseptic meningitis [134]. In a mouse model of cerebral toxoplasmosis, UA treatment notably reduced the number and size of cysts in the brain tissue. Behavioral tests further revealed that UA-treated infected mice exhibited enhanced avoidance behavior towards felid scents, indicating an improved ability to perceive predator risks [135]. Although no studies have yet explored the use of UA in bacterial CNS infections, existing research indicates that UA exhibits substantial antimicrobial activity against various bacteria, including methicillin-resistant Staphylococcus aureus, carbapenem-resistant *Acinetobacter baumannii*, *Campylobacter* species, *Shigella dysenteriae*, and *Vibrio cholerae* [136]. A preclinical study demonstrated that oral UA significantly ameliorated *Campylobacter jejuni* infection in mice, with localized therapeutic effects in the gut, extending to non-gut tissues and systemic benefits [137].

Overall, while the research on UA in CNS infectious diseases is still limited, further in-depth studies are required to comprehensively assess its effects.

### 4.7. CNS Tumors

UA has demonstrated broad-spectrum antitumor activity in several cancer models. In studies on solid tumors, including non-small cell lung cancer, breast cancer, gastric cancer, cholangiocarcinoma, and oral squamous cell carcinoma, UA exerts its antitumor effects through mechanisms such as modulation of antitumor immunity, inhibition of epithelial-mesenchymal transition, cell cycle arrest, induction of endoplasmic reticulum stress, and suppression of the Akt/mTOR signaling pathway [138,139,140,141,142,143,144].

In the realm of CNS tumors, research on UA has primarily focused on glioblastoma (GBM), the most common and aggressive primary brain tumor with a notably low survival rate [145]. In in vitro studies using GBM cell lines, UA induced G0/G1 phase cell cycle arrest, upregulated the SIRT1/FOXO1 signaling axis, and inhibited PI3K/Akt pathway activity in a dose-dependent manner, thereby suppressing tumor cell proliferation [146]. Additionally, the aryl hydrocarbon receptor (AhR) is expressed at higher levels in glioma patients compared to healthy individuals, and UA, as a pharmacological antagonist of AhR, mitigated TNF-α-induced expression of vascular cell adhesion molecule 1 (VCAM-1) and programmed death-ligand 1 (PD-L1) in GBM cells, reversing the immune suppression [147]. In a GBM xenograft mouse model, UA administration also significantly inhibited tumor growth [146,147].

### 4.8. Radiation Brain Injury

RBI is a severe complication that arises in brain tumor patients undergoing radiotherapy, with its pathogenesis closely related to radiation-induced damage to neurons, glial cells, and vascular structures within the CNS [78,79]. In a radiation-induced primary astrocyte model, UA activated the PINK1/Parkin-mediated mitophagy pathway, significantly reducing ROS levels in both cells and mitochondria, restoring mitochondrial morphology and function, and inhibiting the abnormal secretion of vascular endothelial growth factor (VEGF). In co-culture systems, UA treatment alleviated the disruption of endothelial cell tight junction proteins (such as zonula occludens-1 (ZO-1) and claudin) caused by radiation-exposed astrocyte-conditioned medium [148].

### 4.9. Multiple Sclerosis

Multiple sclerosis (MS) is an autoimmune disorder influenced by genetic and environmental factors, characterized by chronic inflammation, demyelination, and neurodegeneration in the CNS [149]. The experimental autoimmune encephalomyelitis (EAE) model, widely utilized due to its pathological similarities to MS, mimics disease progression effectively. In the MOG35-55-induced EAE mouse model, UA targets the AhR to suppress dendritic cell activation and impair antigen presentation. Additionally, UA reduces the expression of Th17 cell-related genes, such as IL-23a, IL-22, and Ccl2, thereby inhibiting Th17 cell differentiation. This action limits the infiltration of pathogenic T cells into the CNS, alleviating inflammation, demyelination, and clinical symptoms in EAE mice [150].

### 4.10. Amyotrophic Lateral Sclerosis

Amyotrophic lateral sclerosis (ALS) is a fatal neurodegenerative disease marked by the progressive degeneration of upper and lower motor neurons, leading to muscle weakness, paralysis, and, typically, death from respiratory failure within three years of symptom onset [151,152]. In a zebrafish ALS model with knocked-down *C9orf72* and expression of glycine–proline dipeptide repeat proteins, UA has been shown to enhance motor performance [153]. Similarly, in the Cu-exposed SOD1(G93A) transgenic mouse model of ALS, UA activates mitophagy, mitigates mitochondrial dysfunction, and reduces neuroinflammation, resulting in improved gastrocnemius muscle integrity and motor function [154].

### 4.11. Spinal Muscular Atrophy

Spinal muscular atrophy (SMA) is an autosomal recessive disorder caused by mutations or deletions in the survival of motor neuron 1 (SMN1) gene, leading to the degeneration of spinal cord anterior horn motor neurons and progressive proximal muscle weakness and atrophy [155]. Research using muscle cells derived from SMA patients demonstrates that UA activates AMPK and enhances autophagic flux, promoting mitochondrial biogenesis and mitophagy [156].

Preclinical evidence for UA’s therapeutic application in CNS disorders is summarized below (Table 1).

## 5. Advances in Clinical Trials of Urolithin A

Although clinical trials targeting UA treatment for CNS disorders have not yet been initiated, multiple clinical trials have demonstrated that UA possesses favorable safety and pharmacokinetic profiles and have validated some of the biological effects observed in in preclinical studies.

Pénélope A. Andreux et al. pioneered the first double-blind, placebo-controlled randomized Phase I clinical trial to evaluate the safety and pharmacokinetic characteristics of UA in elderly subjects in 2016 (ClinicalTrials.gov: NCT02655393) [16]. This study enrolled healthy elderly male and female subjects aged 61 to 85 years. Oral UA demonstrated excellent safety in both single-dose escalation (250–2000 mg) and multiple-dose escalation (250–1000 mg daily for 28 days) cohorts. UA at all tested doses showed good bioavailability in plasma, was unaffected by food intake, and did not accumulate. Additionally, investigators assessed UA distribution in skeletal muscle, detecting UA 8 h after a single oral dose of 2000 mg, primarily in free form, with trace amounts of UA-glucuronide detected in some subjects, while UA-sulfate was undetectable. Biological effect evaluation revealed that in the multiple ascending dose study, subjects in the 500 mg and 1000 mg groups showed dose-dependent reductions in plasma acylcarnitine levels, and skeletal muscle biopsies demonstrated increased mitochondrial genome expression, suggesting UA’s potential to enhance mitochondrial health [16].

Based on UA’s safety and pharmacokinetic data, Sophia Liu et al. conducted a double-blind, placebo-controlled randomized Phase II clinical trial to evaluate UA’s effects on muscle endurance and mitochondrial health in elderly individuals (ClinicalTrials.gov: NCT03283462) [15]. This study enrolled 66 healthy elderly subjects aged 65 to 90 years who received daily oral administration of 1000 mg UA or placebo for 4 months. Results showed that compared to the placebo group, the UA group demonstrated significantly improved muscle endurance in hand and leg skeletal muscles, with significantly reduced plasma levels of mitochondrial health biomarkers (acylcarnitines, ceramides etc.) and inflammatory markers (CRP, etc.) [15]. Anurag Singh et al. conducted a randomized Phase II clinical trial investigating UA’s effects on muscle strength, exercise performance, and mitochondrial health in overweight middle-aged individuals (ClinicalTrials.gov: NCT03464500) [14]. Eighty-eight overweight middle-aged subjects aged 40 to 64 years received daily oral administration of 500 mg UA, 1000 mg UA, or placebo for 4 months. Results indicated that either dose of UA significantly improved leg muscle strength, with subjects in the 1000 mg UA group showing significantly enhanced exercise performance and aerobic endurance. The UA treatment groups exhibited reduced plasma acylcarnitine and CRP levels, with overall reductions in pro-inflammatory cytokines (IFN-γ, IL-1β, TNF-α, etc.). Molecular mechanism studies revealed that muscle RNA sequencing identified significant enrichment of mitochondrial, ribosomal translation, and muscle contraction gene sets in the 500 mg UA group, while the 1000 mg UA group showed no significantly enriched pathways. Proteomic and Western blot analyses demonstrated that UA can affect Parkin-mediated mitophagy markers in skeletal muscle and dose-dependently upregulate mitochondrial tricarboxylic acid cycle and OXPHOS protein levels [14]. Haotian Zhao et al. conducted an 8-week randomized, double-blind, placebo-controlled study on 20 male athletes with resistance training experience. Subjects received daily oral administration of 1000 mg UA, and after 8 weeks, compared to the placebo group, the UA group showed improvements in muscle strength and endurance, with significantly reduced CRP levels [157].

These studies collectively confirm UA’s clinical application potential as a natural mitochondrial health modulator. Multi-phase clinical trials have validated UA’s favorable safety profile and stable pharmacokinetic characteristics, confirming its biological effects in activating mitophagy, improving mitochondrial function, and providing anti-inflammatory benefits. Notably, different doses of UA produce varying effects [14], suggesting that dose selection requires adjustment based on specific pathophysiological requirements. Based on the discovery that UA is primarily distributed in skeletal muscle in free form and exerts mitochondrial health-promoting effects [16], combined with preclinical studies showing that UA primarily crosses the mouse BBB in free form and acts as a direct effector [28], it is speculated that UA may primarily exert its biological effects in free form. Although current research has mainly focused on UA’s effects on the motor system, the established dosing regimen and biological effect mechanisms provide important theoretical foundations and practical references for conducting clinical trials in CNS disorders.

## 6. Future Perspectives and Challenges

Despite extensive research on UA, considerable challenges remain regarding its potential clinical application for CNS disorders, necessitating more comprehensive investigation.

Although previous studies have demonstrated UA’s broad biological activity and complex molecular regulatory pathways, the specific molecular mechanisms underlying its effects remain incompletely understood [158]. Most preclinical studies on UA for CNS disorders have employed monotherapy without comparing efficacy to existing treatments or exploring combinatorial regimens. We propose that future preclinical research should directly compare UA with standard-of-care therapies, investigate synergistic interactions with other drugs, and leverage UA’s unique advantages in improving mitochondrial function to develop combination strategies. Similarly, current preclinical studies primarily utilize oral administration and intraperitoneal routes, with no comparative studies examining therapeutic differences among these administration methods. Furthermore, while current preclinical research demonstrates that UA can cross the BBB [28], brain concentrations reach only approximately 10% of plasma levels, significantly limiting UA’s therapeutic efficacy [27]. Future research should explore novel drug delivery systems to enhance UA brain delivery efficiency, such as nanoparticle-based carriers or prodrug strategies. For example, the previously mentioned PUASi NPs can be specifically recognized and cleaved by esterases to release UA, achieving targeted delivery to SCI injury sites and enhancing UA’s therapeutic efficacy for SCI through ferroptosis inhibition and anti-inflammatory actions [133]. Successful precedents also exist in other therapeutic areas. The integration of UA-loaded hydrogels with polycaprolactone neural guidance conduits has been achieved. This combination promotes axonal regeneration in rat models of sciatic nerve defects through sustained UA release [159]. In summary, future preclinical research should thoroughly investigate urolithin A’s pharmacological effects, toxicological data, drug delivery, and pharmacokinetic properties, providing crucial references for subsequent clinical trials.

Notably, while UA shows therapeutic potential for CNS disorders, existing evidence remains primarily limited to various cellular and animal models, with no related clinical trials conducted. Due to physiological and pathological differences between animals and humans, these disparities may substantially reduce drug efficacy in actual applications or even produce opposite effects. Therefore, conducting clinical trials of UA treatment for CNS disorders is crucial for advancing UA’s practical application. Additionally, while existing clinical trials have demonstrated UA’s favorable safety profile and pharmacokinetic characteristics and explored dose-response relationships, providing theoretical foundations and practical references for clinical trials in CNS disorders, certain limitations persist. Most of these clinical trials were short-term single-agent studies (28 days to 4 months) conducted in healthy populations, leaving gaps in data regarding potential side effects from long-term UA use or interactions with other medications. Given that CNS disorder treatments, particularly for neurodegenerative diseases, require long-term and combination therapy approaches, understanding UA’s long-term safety profile and drug interaction characteristics is essential. Moreover, current clinical trials demonstrate differential effects across various UA dosages [14], making personalized medicine approaches and reliable dosing strategies for UA clinical application worthy directions for future clinical research.

Recent studies have confirmed a close link between gut microbiota and certain CNS disorders, such as AD and PD [160,161]. An individual’s natural UA production is mainly determined by their gut microbiota [17]. Thus, it is worth exploring whether the differences in UA production, which lead to varying UA levels in humans, are causally related to these CNS disorders. Since clinical studies have shown that directly supplementing UA can resolve the differences in natural UA synthesis levels among individuals [22], understanding the relationship between UA and CNS disorders may help us better use UA to prevent certain CNS disorders.

## 7. Conclusions

With global aging trends intensifying, the incidence of CNS disorders continues to rise, posing a significant challenge to public health systems worldwide. Many CNS disorders, such as AD, PD, and brain tumors, currently lack effective treatments. UA, a polyphenolic metabolite derived from gut microbiota processing of dietary ETs and EA, has emerged as a promising candidate for CNS disorders treatment or adjunctive therapy due to its multi-target regulatory properties and low toxicity. This review outlines UA’s in vivo synthesis, pharmacokinetic profile, and key biological effects, including mitophagy, anti-inflammatory, antioxidant, anti-senescence, and anti-apoptosis effects. We systematically summarize preclinical evidence demonstrating UA’s therapeutic potential in CNS disorders. Furthermore, we discuss the latest clinical trials and future challenges associated with UA translation, aiming to advance UA research and application in CNS disorders, offering a promising avenue to improve patient outcomes and mitigate the global burden of these diseases.

## Figures and Tables

**Table 1:** Experimental settings and effects of UA in preclinical studies of central nervous system disorders.

Experimental Model	Route of Administration	Dosage and Duration	UA Effects	References
**Alzheimer’s disease**				
SH-SY5Y cell line transfected with the human APP695 coding region	-	1–10 μM for 24 h	Enhanced mitochondrial biogenesis and mitochondrial respiration	[111]
ReNcell VM cell line transfected with APPSwe plasmids	-	2.5–10 μM for 2 days	Prevented apoptosis and Aβ production Improved autophagy	[112]
Alzheimer’s disease patient iPSC-derived neurons	-	50 μM for 24 h	Increased mitophagy Improved mitochondrial function	[41]
*C. elegans* expressed Aβ or tau	Oral	100 μM daily from eggs to death	Reversed memory impairment Reduced Aβ Increased neuronal mitophagy Improved mitochondrial function	[41]
APP/PS1 mice	Oral	200 mg/kg daily for 2 months	Restorated neuronal mitophagy Reduced Aβ production and neuroinflammation Improved mitochondrial function	[41]
APP/PS1 mice	Intragastric	200 mg/kg daily for 5 months	Improved learning and memory Decreased Aβ accumulation and tau phosphorylation Restored lysosomal functions and mitophagy Decreased DNA damage and neuroinflammation	[105]
3xTgAD mice and 3xTgAD/Polβ^+/−^ mice	Intragastric	200 mg/kg daily for 5 months	Decreased DNA damage and neuroinflammation	[105]
BV2 cell line treated with Aβ	-	0.1–1 μM, pretreatment for 24 h	Reduced inflammation Reduced cell death	[113]
Male C57BL/6J mice with intracerebroventricular injection of okadaic acid	Intraperitoneal	100 mg/kg daily for 20 days	Reversed memory impairment Decreased tau phosphorylation	[113]
HT22 and SH-SY5Y cell line treated with Aβ	-	30 μM for 24 h	Increased autophagy Enhanced Aβ clearance	[114]
Female 3xTgAD	Oral	25 mg/kg daily in alternate weeks for 10 months	Reduces Aβ accumulation Ameliorated memory impairment, associative learning, and exploratory behavior	[114]
5xFAD mice	Intragastric	NA mg/kg for 1 month	Improved social ability	[115]
APP/PS1 mice	Oral	300 mg/kg daily for 2 weeks	Ameliorated cognitive impairment Prevented neuronal apoptosis Enhanced neurogenesis Reduced Aβ accumulation and peri-plaque microgliosis and astrocytosis Reduced neuroinflammation	[61]
HT22 transfected with mutant APP	-	NA μM for 24 h	Improved mitochondrial function	[106]
Homozygous amyloid beta knockin mice	Intraperitoneal	2.5 mg/kg 3 times weekly for 4 months	Ameliorated cognitive impairment Reduced Aβ accumulation Improved mitophagy and mitochondrial function Decreased neuroinflammation	[106]
**Parkinson’s disease**				
Male albino Wistar rats treated with rotenone	Intragastric	Commercial 6-fold concentrated pomegranate juice, 500 mg/kg daily for 45 days	Improved postural stability, olfactory function, and vertical activity Reduced α-synuclein Enhanced neuronal survival Decreased oxidative damage	[27,117]
PC-12 treated with 6-OHDA	-	2.5–10 μM, pretreatment for 2 h	Reduced neurotoxicity Inhibited apoptosis Promoted mitochondrial biogenesis	[118]
Male C57BL/6J mice treated with 6-OHDA	Intraperitoneal	10 mg/kg daily for 1 week	Prevented dopaminergic neurodegeneration Improved mitochondrial dysfunction and damage Promoted neuronal mitochondrial biogenesis	[118]
BV2 cell line treated with Mn	-	10 μM, pretreatment for 2 h	Increased mitophagy Improved mitochondrial dysfunction	[119]
Male C57BL/6J mice treated with Mn	Intraperitoneal	2.3 mg/kg 3 times weekly for 6 weeks	Increased mitophagy Improved mitochondrial dysfunction Improved neurological function Reduced neuroinflammation	[119]
BV2 cell line treated with LPS	-	2.5–10 μM, pretreatment for 2 h	Suppressed inflammatory response Increased mitophagy Improved mitochondrial dysfunction and mitochondrial metabolism	[120]
Mice injected with MPTP	Intraperitoneal	20 mg/kg daily for 1 week	Ameliorated motor deficits and dopaminergic neurodegeneration Increased autophagy Reduced neuroinflammation	[120]
BV2 cell line with MPP^+^ stimulation	-	2.5–5 μM, pretreatment for 1 h	Suppressed inflammatory response Increased mitophagy Improved DA neuron injury	[121]
Male C57BL/6J mice injected with MPTP hydrochloride	Intragastric	200 mg/kg daily for 11 days	Increased mitophagy Reduced neuroinflammation Improved DA neuron injury	[121]
**Diabetes-associated cognitive impairment**				
HT22 cell line treated with DMNQ	-	2.5–10 μM, pretreatment for 3 h	Inhibited ER stress, and apoptosis Decreased oxidative damage Improved mitochondrial function Attenuated calcium overload and disorder	[125]
Mouse primary hippocampal neurons treated with 45 mM glucose	-	5 μM for 24 h	Inhibited tau hyperphosphorylation, ER stress, and apoptosis	[125]
Male C57BL/6J mice treated with HFD and STZ	Intragastric	200 mg/kg daily for 10 weeks	Improved hyperglycemia Ameliorated cognitive impairment Attenuated systemic inflammation Ameliorated the intestinal barrier dysfunction	[124,125]
SH-SY5Y cell line treated with a high glucose or Aβ	-	0.1 μM, pretreatment for 48 h	Decreased mitochondrial calcium influx, and mtROS accumulation Reduced Aβ production	[123]
iPSC-derived neurons treated with a high glucose	-	0.1 μM, pretreatment for 0.5 h	Decreased mitochondrial calcium influx, and mtROS accumulation Reduced Aβ production	[123]
Male CrljOri:CD1(ICR) mice treated with STZ	Intraperitoneal	2.5 mg/kg daily for 8 weeks	Improved cognitive impairment Decreased Aβ production and tau phosphorylation	[123]
HT22 treated with high glucose and palmitate	-	100 μM for 24 h	Increased mitophagy	[46]
Male C57BL/6J mice treated with HFD and STZ	Intraperitoneal	2.5 mg/kg daily for 8 weeks	Improved learning and memory Increased mitophagy Improved mitochondrial function	[46]
**Ischaemic stroke**				
Mouse primary cortical neuronal and N2a cell line, oxygen-glucose deprivation/reperfusion model	-	3–30 μM for 1 h	Inhibited neuronal injury Induced autophagy Repressed ER stress	[128]
Male C57BL/6 mice with middle cerebral artery occlusion	Intraperitoneal	2.5–5 mg/kg for 24 h and 1 h prior to operation	Reduced acute ischemic brain injury Induced autophagy Repressed ER stress	[128]
Male C57BL/6 mice with middle cerebral artery occlusion	Intraperitoneal	1.5–2 mg/kg at 1 h and 24 h prior to operation 1.5–2 mg/kg after surgery	Improved neurological outcomes Inhibited neuronal injury and apoptosis Reduced neuroinflammation	[99]
**Subarachnoid hemorrhage**				
Wistar rats, endovascular perforation Subarachnoid hemorrhage model	Intraperitoneal	2.5–10 mg/kg at 30 min prior to operation	Relieved neurological deficits, BBB disruption, and cerebral edema Inhibited neuronal apoptosis Improved autophagy	[129]
**Traumatic brain injury**				
Male C57BL/6J mice, controlled cortical impact model	Intraperitoneal	2.5–10 mg/kg daily for 3 days	Ameliorated neurological deficits Relieved BBB disruption, and cerebral edema Attenuated neuronal apoptosis Promoted neuronal autophagy	[98]
**Spinal cord injury**				
BV2 treated with LPS and ATP	-	5–20 μM, pretreatment for 2 h	Inhibited pyroptosis and inflammatory response Improved mitophagy and mitochondrial homeostasis	[132]
Female C57BL/6 mice with striking the spinal cord	Intragastric	50 mg/kg daily for 8 weeks	Attenuated motor dysfunction Promoted autophagy Decreased pyroptosis	[132]
PC-12 treated with RSL3	-	PUASi NPs, 30 μM for 6 h	Inhibited ferroptosis	[133]
BV2 treated with LPS or LPS and IFN-γ	-	PUASi NPs, 30 μM for 24 h	Inhibited M1 polarization and inflammatory response	[133]
Female C57BL/6 mice with striking the spinal cord	Intraperitoneal	PUASi NPs, 10 mg/kg daily for 7 days	Attenuated motor dysfunction Reduced neuroinflammation and neuronal damage Promoted nerve regeneration Inhibited ferroptosis	[133]
**EV71 infection**				
SK-N-SH cell line infected with EV71	-	25 μM for 12 h and 24 h	Inhibited virus proliferation	[134]
**Cerebral toxoplasmosis**				
Differentiated ReNcell infected with *T. gondii*	-	50–100 μM for 24 h	Reduced tachyzoite load and perturbs cyst formation	[135]
Female BALB/cJInv mice infected with *T. gondii*	Intraperitoneal	30 μg daily for 34 days	Increased survival Inhibited cyst formation Improved the innate response towards predatory cat odor	[135]
**Glioblastoma**				
U251, U118, and U87 cell line	-	5–80 μM	Inhibited migration ability and cell proliferation	[146]
Male BALB/c nu/nu mice, xenograft model	Intragastric	50 mg/kg daily for 15 days	Suppressed the tumor growth	[146]
U251, U87, and ALTS1C1 cell line	-	2–10 μM	Inhibited migration ability and cell proliferation Reduce expression of VCAM-1 and PD-L1	[147]
Male C57BL/6 mice, xenograft model	Intraperitoneal	40 mg/kg for 9 days	Suppressed the tumor growth	[147]
**Radiation brain injury**				
Mouse primary astrocytes received 20 Gy of irradiation	-	10 μM for 24 h	Improved mitophagy and mitochondrial homeostasis Reduced BBB injury	[148]
**Multiple sclerosis**				
Female C57BL/6 mice, EAE model	Oral	25 mg/kg on the day of immunization, disease onset, and peak respectively	inhibited myelin depletion Reduced neuroinflammation Inhibited migration of pathogenic T cells from the periphery to CNS Suppressed the activation of dendritic cells Restricted Th17 polarization	[150]
**Amyotrophic lateral sclerosis**				
Zebrafish expressed glycine–proline DPR in a *C9orf72* knockdown context	embryo medium containing urolithin A	5 μM	Improved the swimming ability	[153]

Abbreviations: 6-OHDA, 6-hydroxydopamine; ATP, adenosine triphosphate; Aβ, amyloid β; BBB, blood–brain barrier; CNS, central nervous system; DA, dopaminergic; DMNQ, 2,3-dimethoxy-1,4-naphthoquinone; DPR, dipeptides repeats; EAE, experimental autoimmune encephalomyelitis; ER, endoplasmic reticulum; EV71, enterovirus 71; HFD, high-fat diet; Mn, manganese; IFN-γ, interferon gamma; iPSC, pluripotent stem cell; LPS, lipopolysaccharide; MPP+, 1-methyl-4-phenylpyridinium ion; MPTP, 1-methyl-4-phenyl-1,2,3,6-tetrahydropyridine; mtROS, mitochondrial reactive oxygen species; NA, unknow; PD-L1, programmed cell death ligand 1; PUASi NPs, PEGylated UA-silicon hybrid nanoparticles; RSL3, ferroptosis activator; STZ, streptozotocin; Th17, T helper cell 17; VCAM-1, vascular cell adhesion molecule-1.
